# Immuno-Detection of C3a, a C3 Complement Activated Product in Mastitis Milk, a Potential Diagnostic Marker

**DOI:** 10.3390/vetsci4010013

**Published:** 2017-02-23

**Authors:** Thanislass Jacob, Gangasudan Subramani, Prathiba Sivaprakasam, Antony P. Xavier, Hirak K. Mukhopadhyay

**Affiliations:** 1 Department of Veterinary Biochemistry, Rajiv Gandhi Institute of Veterinary Education and Research, Puducherry 605 009, India; gangasudan@gmail.com (G.S.); prathi.10@gmail.com (P.S.); 2 Department of Veterinary Microbiology, Rajiv Gandhi Institute of Veterinary Education and Research, Puducherry 605 009, India; pxantony@gmail.com (A.P.X.); mhirak@rediffmail.com (H.K.M.)

**Keywords:** mastitis, bio-marker, acute phase protein, complement component C3

## Abstract

The sub-clinical form of mastitis is difficult to detect and causes huge economic loss to the dairy industry. It has become a threat to public health at large, thus there is a need for definite diagnosis of the disease. Therefore, this study was undertaken to identify the novel diagnostic marker for the detection of the sub-clinical form of mastitis. Two-dimensional gel analysis of the whey protein fraction of normal and mastitis milk samples revealed the presence of proteose peptone component 3 precursor, Trypsin precursor, complement component-C3, Ig heavy chain precursors and a C-type lectin domain as differentially expressed protein during the early stage of mastitis. Of these proteins identified, complement component-C3 was tested for its diagnostic potential. Western blot analysis of the milk whey of sub-clinical mastitis cases (M+, M++ & M+++) identified the accumulation of C3a, an activated product of complement component-C3. Further, the hemolytic activity of the above milk whey samples positively correlated with the somatic cell count. As C3a is already reported as an anaphylotoxic agent, it chemo tactically attracts lymphocytes at the site of inflammation, the detection of which in the milk whey can be of diagnostic importance for sub-clinical mastitis.

## 1. Introduction

Mastitis is one of the most important diseases of dairy cattle worldwide. Within the mastitis condition, sub-clinical mastitis is of greater importance because of its adverse effect on milk production and quality and its impact on public health. The prevalence of clinical mastitis in dairy herds in Europe is usually below 55%, whereas the prevalence of sub-clinical intra-mammary infection normally lies between 20% and 30%, but can range from 7% to greater than 60% [1]. According to a study undertaken in India by Joshi and Gokhale [2], the prevalence of sub-clinical mastitis is greater than the prevalence of clinical mastitis. Sub-clinical intra-mammary infections are, in general, poorly detected and can lead to persistent udder infection [3,4]. The diagnosis of sub-clinical mastitis is more problematic than the diagnosis of clinical mastitis. In spite of extensive research in the field of mastitis, Somatic Cell Count (SCC) in combination with the microbial culture technique is the gold standard for the diagnosis of sub-clinical mastitis. In addition, indirect assays such as the California Mastitis test (CMT) and the Electrical Conductivity test are still being practiced extensively. However, all these tests suffered from their own limitations [5,6,7]. Therefore, now the focus of the research on mastitis diagnosis has shifted towards the identification of protein biomarkers which can be used as an indicator of inflammation, trauma and other pathological conditions. At the outset of the 21st century, research on the diagnosis of mastitis focused on measuring acute phase proteins (APPs) in milk and on determining the factors that contributed to their presence [8]. The reports of APPs produced in the mammary gland in response to experimental and natural infections with the bacteria that cause bovine mastitis have led to the exciting potential of APPs being used in the detection of this economically important disease [9,10].

Proteomics is the approach which is being widely used to identify and characterize the clinically useful biomarkers. These biomarkers thus identified have potential as diagnostic markers and can also be used to study the patho-physiology of the disease. Therefore, the proteomic approach was applied to detect the novel protein associated with the early stage of mastitis, and biomarkers thus identified were further validated with special reference to complement component-C3 as a possible diagnostic marker for the sub-clinical form of mastitis.

## 2. Materials and Methods

### 2.1. Sample Collection

About 10–15 mL of milk was collected from each quarter (Right Fore Quarter, Right Hind quarter, Left Fore Quarter and Left Hind Quarter) of the udder of a cow. A total of 126 milk samples were collected from two different farms located in and around Puducherry, India. The milk samples were collected simultaneously and subjected to a California Mastitis Test (CMT) and somatic cell count testing. Based on the CMT results, milk samples were categorized as normal (13 no.), M+ (46 no.), M++ (30 no.) and M+++ (37 no.). The result of M+ and M++ was considered as sub clinical mastitis as it was found to associate with a somatic cell count of ≤1,200,000.

### 2.2. Microbial Examination

Nutrient agar supplemented with 5% sterile defibrinated ovine blood and Mac Conkey’s agar was used for the isolation of microorganisms. Milk samples were mixed well and two to three loopfuls of milk were streaked onto blood agar and Mac Conkey’s agar plates and incubated at 37 °C for 24–48 h. A minimum of five colonies of the same type were recorded as the causative agent and more than one type of colony was determined as mixed growth of infection. Out of the milk samples (113 no.) which were found to be positive for CMT, 68 samples were culturally positive. Of the sixty-eight, 27 samples were only positive for *S. aureus* which were subjected for proteomic analysis. The rest of the samples were found to be positive for *E. coli*, *Staphylococcus*, *Klebsiella* sp., yeast, either individually or in mixture. 

### 2.3. Hemolytic Titre

This is the measure of the complement and its activation. All the 68 sub-clinical mastitis milk samples were tested for the presence of haemolytic activity. Sheep Red Blood Corpuscles (RBCs) were lysed by the complement components present in the milk at different titer levels. It is correlated with the SCC. 

### 2.4. Removal of High Abundance Proteins from Milk

The mastitis milk samples which are only positive for *S. aureus* were used for further analysis. The milk samples were transferred to clean centrifuge tubes and were subjected to centrifugation at 4 °C, 6000× *g* for 20 min to remove the fat which accumulates as a layer at the top of the milk sample.

The major interfering protein, namely Casein, was precipitated from the de-fattened milk fractions through the “salting out” technique using 35% Ammonium Sulphate saturation (19.4 g per 100 mL) as described by Hogarth et al. [11]. The whey protein, free from the casein protein, was separated as supernatant by centrifugation at 4 °C, 6000× *g* for 20 min and subjected to dialysis for the removal of salts. Then, total protein was measured using Lowry’s method [12]. 

### 2.5. SDS-PAGE Analysis of Whey Proteins

**Figure 1:**
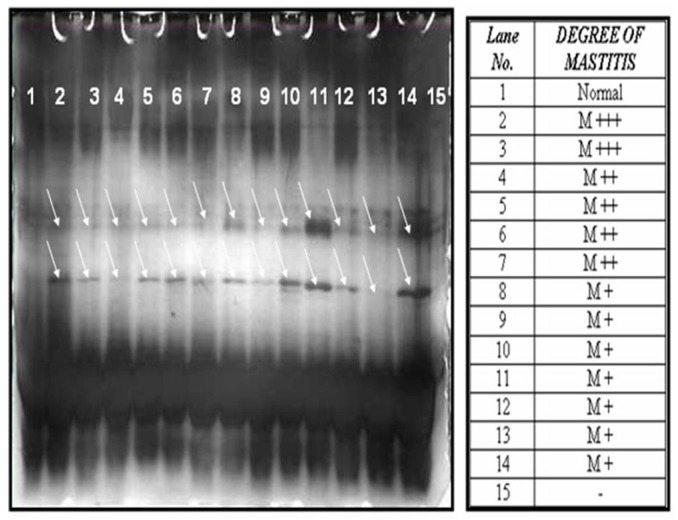
The 12% SDS-PAGE analysis of the whey protein of bovine milk (normal and mastitis milk) and silver staining.

The whey protein prepared from the normal, as well as mastitis milk samples with the same concentration of protein were subjected to 16% SDS-PAGE (26 × 20 cm) at 160 volts for 6 h. The gels were silver stained [13] to visualize the protein bands. The banding pattern obtained was compared between the normal and mastitis condition and it was found that there were few bands which appeared at the molecular range of around 10 to 26 kda, only in the case of mastitis milk samples, as shown in Figure 1.

### 2.6. Two-Dimensional (2D) Gel Electrophoresis

Six samples (three normal and three mastitis) which were analyzed by SDS-PAGE and which had shown distinct sharp bands without any smear were selected and subjected to 2D gel analysis [11].

**Figure 2:**
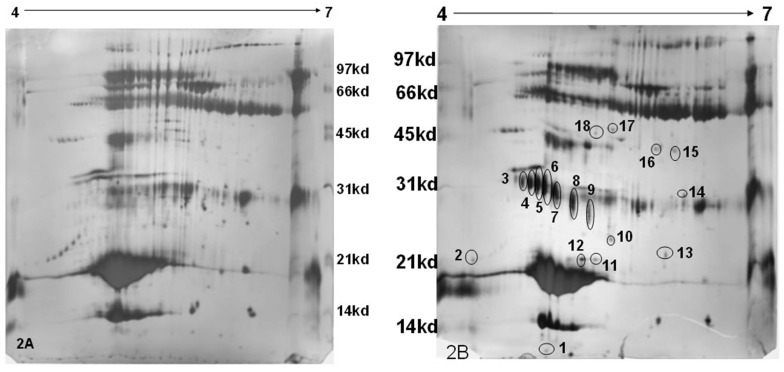
Two-dimensional (2D) gel electrophoresis of the whey protein of bovine milk and silver staining ((2A)—normal milk; (2B)—mastitis milk (M+)). Mastitis milk samples show differentially expressed proteins (1 to 18 spots).

Two-hundred micro-liters of the Rehydration solution was mixed with 50 µL of the sample (150 µg). This was transferred to the strip holder and the gel strip (pH 4 to 7 (L), 18 cm in length) was placed with the gel side facing downward such that the entire gel comes into contact with the solution. In order to prevent the evaporation of the Rehydration solution, about 1 mL of immobiline dry strip cover fluid (GE Healthcare Bio-sciences AB, Uppsala, Sweden) was added over the gel that was placed over the Rehydration-sample mixture. This setup was left undisturbed over-night. The rehydrated gel strip was subjected to Iso-Electric Focusing using the following voltage protocol: 50 V, 500 Vh; 500 V, 250 Vh; 2000 V, 1000 Vh; 5000 V, 5000 Vh; 8000 V, 8000 Vh; 10,000 V, 60,000 Vh. The first dimensional run was for about 20 h. This was followed by two equilibration steps, firstly with Dithiotheritol (DTT) and secondly with Iodoacetamide. A 12% resolving gel (26 × 20 cm) was cast and the equilibrated Immobiline Dry Strip gel (IPG) strip was placed horizontally. Over this agarose embedding solution, acting as the sealing solution, was added. The second dimension was run for 7 h at 200 V at 25 °C. The gel was stained using silver nitrate as described above. The developed gels are shown in Figure 2a,b. 

The position and intensity of individual spots were detected using image master 2D platinum 6.0 software (GE Healthcare Biosciences AB, Uppsala, Sweden). The differentially expressed protein was detected based on the changes in stain density and intensity of two-fold or more.

### 2.7. LC-MS/MS Analysis

The differentially expressed protein spots (18 no.) were extracted and subjected to in-gel tryptic digestion. The gel pieces were vacuum dried and rehydrated with trypsin (Promega) in 50 mM ammonium bicarbonate (pH 7.8). The mixture was incubated overnight at 37 °C with constant shaking. The liberated peptides were extracted with 25 mM ammonium bicarbonate (pH 7.8) and further extracted with the solution containing 0.25% (*v/v*) Trifluoroaceteic anhydride (TFAA) and 50% (*v/v*) actetonitrile. The extract was dried and resuspended in 50% acetonitrile/5% formic acid for the ion trap MS analysis.

The peptide mixture (20.0 µL) at the concentration of 100 µg/mL was injected onto a BioBasic C-18 column (150 × 0.18 cm) with a flow rate of 120 µL/min. The column temperature was maintained at 30 °C. The liquid chromatography column was equilibrated using Millipore water/0.1% formic acid and subjected to gradient elution using aceteonitrile/0.1% formic acid at a flow rate of 200 µL/min. The peptide mass was determined on an ion trap mass spectrometer (Thermo Fischer, San Jose, CA, USA), where the capillary temperature was maintained at 250 °C. The electrospray voltage was set to 45 KV, the capillary voltage at 22 V, and the tube lens offset at −6 V. In the full scan mode, ions were collected in three micro-scans with a maximum ion injection time of 200 ms; MS spectra for all samples were measured with an overall mass/charge (*m/z*) range of 400 to 2000; MS/MS was carried out in the data-dependent mode. Peptides were characterized using SEQUEST software (Bioworks 2.0, Thermo Fischer, San Jose, CA, USA), which used the tandem mass spectra of peptide ions to search against the mammals’ taxonomy of the Swissprot/Mass spectrometry sequence database(MSDB), protein database. The identification of the protein was done using the MASCOT search engine (www.matrixscience.com). The search parameters employed were as follows—Type of search: Peptide Mass fingerprint; fixed modifications: Cabamidomethyl for cysteine; Variable modifications: Oxidation for methionine; Mass values: Monoisotopic; Protein Mass: Unrestricted; Peptide mass tolerance: ±100 ppm; Peptide Charge state: 1+; Max missed cleavages: 1; Number of queries: 49; Selected for scoring: 25. 

### 2.8. Western Blot Analysis

Milk whey proteins (10 µg) from normal and mastitis (M+, M++ & M+++) conditions were electrophoretically resolved on a 12% SDS-PAGE (125 V for 1.5 h) and electro blotted (50 V for 1 h) onto a nitrocellulose paper. The blot was probed with a rabbit polyclonal primary antibody against complement component-C3 (Abcam, Cambridge, UK) as it was reported to have cross reactivity with bovine complement, and developed with HRP-DAB system. The procedure was repeated with three different samples in each condition. 

## 3. Results and Discussion

MASCOT analysis of Peptide Mass Fingerprints of mastitis milk resulted in the identification of proteose peptone component 3 precursor (PP3), Trypsin precursor, complement component-C3, Ig heavy chain precursor, C-type lectin domain and β-lactoglobulin, one of the major milk proteins (Figure 2a,b and Table 1). The spots related to trypsin precursor and PP3 were detected at different locations on 2D gel with difference in pI and Molecular Weight (MW). This could be due to the formation of isoforms resulted out of the difference in glycosylation and/or the phosphorylation of proteins. 

Among the proteins identified, PP3, C-type lectin domain and complement component-C3 are known to be associated with the infectious disease process. The PP3 content in milk with a high SCC is correlated with the intensity of proteolytic activity, so PP3 fraction is widely used as an indicator of caseinolysis [14,15]. Component-3 of proteose peptone (PP3), also called lactophorin, is a minor phosphoglycoprotein (135 residue) [16] found in bovine milk. Considering the pore forming ability of the 113 to 135 C-terminus peptide of bovine PP3, it was conceivable that this peptide could interact with a natural lipids bilayer, such as bacterial membrane, and it was demonstrated that this peptide displayed inhibitory growth activity against Gram-positive and Gram-negative bacteria but was inefficient to induce hemolysis of human red blood cells [17]. Therefore, it is likely that the expression of this protein may be enhanced at the time of infection.

The complement system plays a major role in the host defense mechanisms against infectious microbes, as it is involved in both specific and non-specific immunity. Apart from serum, the complete complement system can be found in bovine colostrum, and components of the system are also present in milk.

The source of complement component-C3 found in bovine milk is varied and it may be derived from the transduction or exudation or local synthesis. Rainard [18] suggested that the milk complement had partly different origins as a function of the health status of the mammary gland. Transudation and local synthesis of C3 may dominate in normal milk, whereas exudation and stimulated local synthesis are likely to dominate during infection.

**Figure 3:**
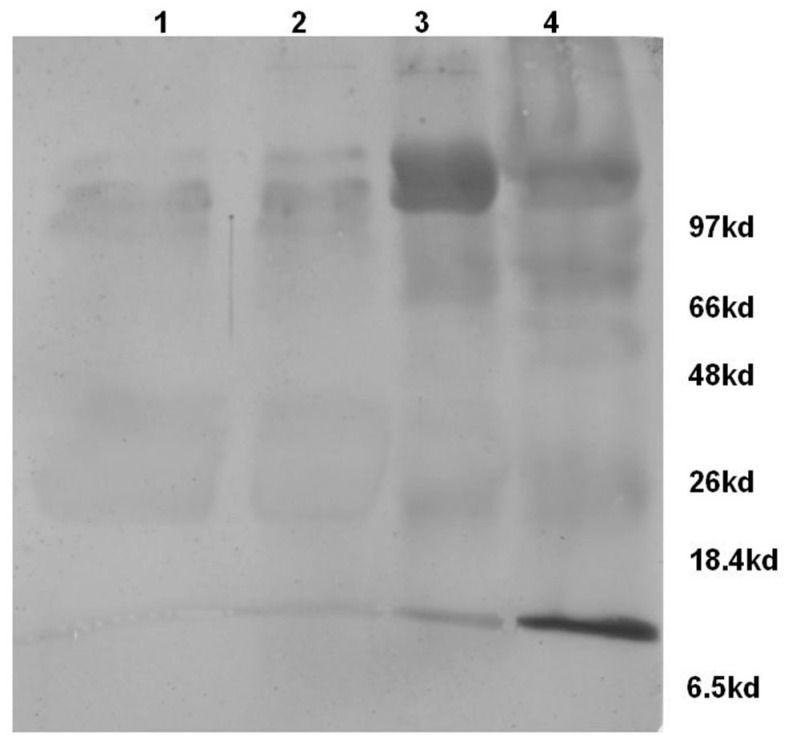
Western blot analysis of bovine milk whey proteins (Lane 1—Normal; Lane 2—Mastitis (M+); Lane 3—Mastitis (M++); Lane 4—Mastitis (M+++)) for the presence of complement component—C3 and its activated products. Mastitis milk samples show the presence of α-chain of C3b (~105 kda), β-chain of C3 (~75 Kda) and C3a (~9 Kda).

Therefore, the milk samples categorized based on CMT were subjected to Western blot analysis using Rabbit polyclonal antibodies against human complement component-C3. The analysis revealed the presence of complement component-C3 in both normal as well as mastitis milk samples. However, the levels were enhanced in the mastitis condition, in particular M++ and M+++ categories. Further, the blot (Figure 3) also revealed the presence of the activated product of complement component-C3, i.e., α-chain of C3b (105 KDa) and C3a (9 KDa) protein which confirms the activation of complement component-C3 in the mastitis milk.

**Figure 4:**
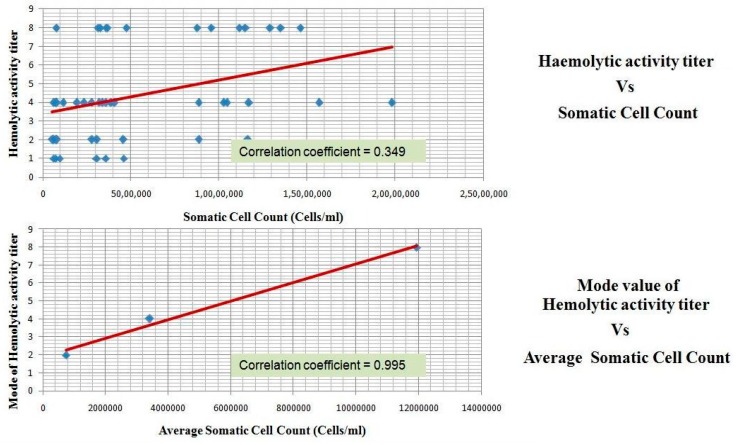
Haemolytic activity of milk whey samples and its correlation with somatic cell count.

Complement is activated during infection and results in the formation of biologically active peptides C5a and C3a which elicit a number of proinflammatory effects, such as the chemotaxis of leukocytes, the degranulation of phagocytic cells, mast cells, and basophils, smooth muscle contraction, and increase of vascular permeability [19]. Therefore, it can be construed that the accumulated complement activated product-C3a observed in this study could have led to the pro-inflammatory effects reported in the mastitis, for example, the increase in the hemolytic activity of milk whey positively correlated with the increase in somatic cell count (Figure 4).

At present, attention is being focused on using acute phase proteins such as haptoglobulin, serum amyloid A, etc., as bio-markers for the diagnosis of mastitis, but they are termed as non-specific markers of the inflammatory process. Therefore, use of complement component may be considered for the diagnosis of mastitis as it was proved to be directly related to the elimination of microorganisms that are being activated during infection and the activated products such as C3a are detected in the milk, as demonstrated in this study. 

## Figures and Tables

**Table 1:** MASCOT search results of peptide mass fingerprints.

Spot No.	Name of the Protein	Mass	Protein Score	Peptide
1 and 8	Trypsin Precursor	25,078	84	R.VATVSLPR.S
				R.VAVSLPR.S
				R.VATVSLPR.S
				K.LSSPATLNSR.V
				K.APVLSDSSCKSSY
2 to 7	Proteose peptone component 3 precursor	17,198	79	K.EQIVIR.S
				K.LPLSILK.E
				P.NLENTVK.E
				K.LMELGHK.I
				K.SLFSHAFEVVK.T
				K.SLFSHAFEVVK.T
11 and 12	β-Lactoglobulin	18,541	66	K.IPAVFK.I
				K.IDALNENK.V
				-.IIVTQTMK.G
				-.IIVTQTMK.G
				-.IIVTQTMK.G
				K.VLVLDTDYK.K
				R.TPEVDDEALEK.F
13	C-type lectin domain	22,586	53	K.MLEELK.T
				K.EQQALQTVCLK.G
14	Complement component-C3	188,229	67	K.VVPEGVR.V
				R.LPYSVVR.N
				K.LMNVFLK.D+Ox
				R.AILYNYR.E
				K.IGLHEVEVK.A
				R.HQQTITIPAR.S
15	Ig Heavy chain precursor	51,391	77	K.VHNEGLPAPIVR.T
				R.EPQVYVLAPPQEEL
17	AMO86793 NID: Bos Taurus	188,715	124	K.KFDLR.V
				K.ISYIIGK.D
				K.ISYIIGK.D
				K.TLSTGVDR.Y
				K.EVTLEDR.L
				R.VSIRPAPETVK.K
				K.EVTLEDRLDK.A
